# Multi-arm Trial of Inflammatory Signal Inhibitors (MATIS) for hospitalised patients with mild or moderate COVID-19 pneumonia: a structured summary of a study protocol for a randomised controlled trial

**DOI:** 10.1186/s13063-021-05190-z

**Published:** 2021-04-12

**Authors:** Nikhil Vergis, Rachel Phillips, Victoria Cornelius, Alexia Katsarou, Taryn Youngstein, Lucy Cook, Michelle Willicombe, Clio Pilay, Tina Shturova, Melanie Almonte, Asad Charania, Richard Turner, Onn Min Kon, Graham Cooke, Mark Thursz, Svetlana Cherlin, James Wason, Dragana Milojkovic, Andew J. Innes, Nichola Cooper

**Affiliations:** 1 Imperial College, London and Imperial NHS Trust, London, UK; 2 Imperial Clinical Trials Unit and Imperial College, London, UK; 3 Newcastle Clinical Trials Unit and Newcastle University, Newcastle upon Tyne, UK

**Keywords:** COVID-19, coronavirus, randomised controlled trial, protocol, pneumonia, fostamatinib, ruxolitinib

## Abstract

**Objectives:**

The primary objective of MATIS is to determine the efficacy of ruxolitinib (RUX) or fostamatinib (FOS) compared to standard of care (SOC) with respect to reducing the proportion of hospitalised patients progressing from mild or moderate to severe COVID-19 pneumonia.

Secondary objectives, at 14 and 28 days, are to:

- Determine the efficacy of RUX or FOS to reduce mortality
- Determine the efficacy of RUX or FOS to reduce the need for invasive ventilation or ECMO
- Determine the efficacy of RUX or FOS to reduce the need for non-invasive ventilation
- Determine the efficacy of RUX or FOS to reduce the proportion of participants suffering significant oxygen desaturation
- Determine the efficacy of RUX or FOS to reduce the need for renal replacement therapy
- Determine the efficacy of RUX and FOS to reduce the incidence of venous thromboembolism
- Determine the efficacy of RUX and FOS to reduce the severity of COVID-19 pneumonia [graded by a 9-point modified WHO Ordinal Scale*
- Determine the efficacy of RUX or FOS to reduce systemic inflammation
- Determine the efficacy of RUX or FOS to the incidence of renal impairment
- Determine the efficacy of RUX or FOS to reduce duration of hospital stay
- Evaluate the safety of RUX and FOS for treatment of COVID-19 pneumonia.

**Trial design:**

A multi-arm, multi-stage (3-arm parallel-group, 2-stage) randomised controlled trial that allocates participants 1:1:1 and tests for superiority in experimental arms versus standard of care.

**Participants:**

Patients will be recruited while inpatients during hospitalisation for COVID-19 in multiple centres throughout the UK including Imperial College Healthcare NHS Trust.

INCLUSION:

- Patients age ≥ 18 years at screening
- Patients with mild or moderate COVID-19 pneumonia, defined as Grade 3 or 4 severity by the WHO COVID-19 Ordinal Scale
- Patients meeting criteria:
  - Hospitalization *AND*
  - SARS-CoV2 infection (clinically suspected or laboratory confirmed) *AND*
  - Radiological change consistent with COVID-19 disease
- CRP ≥ 30mg/L at any time point
- Informed consent from patient or personal or professional representative
- Agreement to abstain from sexual intercourse or use contraception that is >99% effective for all participants of childbearing potential for 42 days after the last dose of study drug. For male participants, agreement to abstain from sperm donation for 42 days after the last dose of study drug.

EXCLUSION:

- Requiring either invasive or non-invasive ventilation including CPAP or high flow nasal oxygen at any point after hospital admission but before baseline, not related to a pre-existing condition (e.g., obstructive sleep apnoea)
- Grade ≥ 5 severity on the modified WHO COVID-19 Ordinal Scale, i.e. SpO_2_ < 90% on ≥ 60% inspired oxygen by facemask at baseline; non-invasive ventilation; or invasive mechanical ventilation
- In the opinion of the investigator, progression to death is inevitable within the next 24 hours, irrespective of the provision of therapy
- Known severe allergic reactions to the investigational agents
- Child-Pugh B or C grade hepatic dysfunction
- Use of drugs within the preceding 14 days that are known to interact with any study treatment (FOS or RUX), as listed in the Summary of Product Characteristics
- Pregnant or breastfeeding
- Any medical condition or concomitant medication that in the opinion of the investigator would compromise subjects’ safety or compliance with study procedures.
- Any medical condition which in the opinion of the principal investigator would compromise the scientific integrity of the study

Non-English speakers will be able to join the study. If participants are unable to understand verbal or written information in English, then hospital translation services will be requested at the participating site for the participant where possible.

**Intervention and comparator:**

RUXOLITINIB (RUX) (14 days): An oral selective and potent inhibitor of Janus Associated Kinases (JAK1 and JAK2) and cell proliferation (Verstovek, 2010). It is approved for the treatment of disease-related splenomegaly or constitutional symptoms in myelofibrosis, polycythaemia vera and graft-versus-host-disease. RUX will be administered orally 10mg bd Day 1-7 and 5mg bd Day 8-14.

FOSTAMATINIB (FOS) (14 days): An oral spleen tyrosine kinase inhibitor approved for the treatment of thrombocytopenia in adult participants with chronic immune thrombocytopenia. FOS will be administered orally 150mg bd Day 1-7 and 100mg bd Day 8-14. Please see protocol for recommended dose modifications where required.

COMPARATOR (Standard of Care, SOC): experimental arms will be compared to participants receiving standard of care. It is accepted that SOC may change during a rapidly evolving pandemic. Co-enrolment to other trials and rescue therapy, either pre- or post-randomisation, is permitted and will be accounted for in the statistical analysis.

**Main outcomes:**

Pairwise comparison (RUX vs SOC and FOS vs SOC) of the proportion of participants diagnosed with severe COVID-19 pneumonia within 14 days. Severe COVID-19 pneumonia is defined by a score ≥ 5 on a modified WHO COVID-19 Ordinal Scale, comprising the following indicators of disease severity:

- Death *OR*
- Requirement for invasive ventilation *OR*
- Requirement for non-invasive ventilation including CPAP or high flow oxygen *OR*
- O_2_ saturation < 90% on ≥60% inspired oxygen

**Randomisation:**

Participants will be allocated to interventions using a central web-based randomisation service that generates random sequences using random permuted blocks (1:1:1), with stratification by age (<65 and ≥65 years) and site.

**Blinding (masking):**

No participants or caregivers are blinded to group assignment. Clinical outcomes will be compared blind to group assignment.

**Numbers to be randomised (sample size):**

For an early informal dose examination by the Data Monitoring Committee a minimum of 30 participants will be recruited.

For Stage 1 of this multi-arm multi-stage study, 171 participants will be randomised, with 57 participants in each arm.

If at least one experimental intervention shows promise, then Stage 2 will recruit a further 95 participants per arm. Sample size calculations are given in the protocol.

**Trial Status:**

Recruitment is ongoing and started 2^nd^ October 2020. We anticipate completion of Stage 1 by July 2021 and Stage 2 by April 2022. The current protocol version 2.0 of 11^th^ February 2021 is appended.

**Trial registration:**

EudraCT: 2020-001750-22, 9^th^ July 2020

ClinicalTrials.gov: NCT04581954, 9^th^ October 2020

**Full protocol:**

The full protocol is attached as an additional file, accessible from the Trials website (Additional file 1). In the interest of expediting dissemination of this material, familiar formatting has been eliminated; this Letter serves as a summary of the key elements of the full protocol.

**Supplementary Information:**

The online version contains supplementary material available at 10.1186/s13063-021-05190-z.

## Supplementary Information

**Additional file 1.**

## Data Availability

Not applicable

