# Supplementary material for: Multi-arm Trial of Inflammatory Signal Inhibitors (MATIS) for hospitalised patients with mild or moderate COVID-19 pneumonia: a structured summary of a study protocol for a randomised controlled trial
Source: Trials. 2021 Apr 12;22:270. doi: 10.1186/s13063-021-05190-z (PMC8039797; doi:10.1186/s13063-021-05190-z)
Supplement: Supplementary file 1 — Additional file 1. [file 13063_2021_5190_MOESM1_ESM.pdf]

Imperial College London

Imperial College Healthcare NHS Trust

Multi-arm trial of Inflammatory Signal Inhibitors for COVID-19 (MATIS)

Sponsor:	Imperial College London
Funder:	Imperial Biomedical Research Centre
Study coordination centre:	
Sponsor Protocol Number:	20HH5926
IRAS ID:	282552
EudraCT:	2020-001750-22
IRAS Project ID:	282552
REC reference:	20/HRA/2618
Protocol Version:	2.0
Protocol Date	11th February 2021
Study Product	ruxolitinib (RUX); fostamatinib(FOS)
Key Words:	coronavirus, COVID-19, pneumonia, ruxolitinib, fostamatinib

PROTOCOL AUTHORISED BY

Name: Dr Nichola Cooper **Role**: Chief Investigator

Signature: ____________________ **Date**: ____________________

ROLES AND RESPONSIBILITIES

These membership lists are correct at the time of writing; please see terms of reference documentation in the Trial Master File (TMF) for current lists.

TRIALTEAM

Name	Affiliation	Role	Contact details
Dr Nichola Cooper	Imperial College Healthcare NHS Trust, Hammersmith Hospital, London UK	Chief Investigator	n.cooper@imperial.ac.uk<br>Tel: 07766546432
Dr Nikhil Vergis	Imperial College Healthcare NHS Trust, St Mary's Hospital (SMH), London UK	Principal Investigator SMH	n.vergis@imperial.uk
Dr Anna Daunt	Imperial College Healthcare NHS Trust, St Mary's Hospital, London UK	Co-Investigator	a.daunt@nhs.net
Dr Richard Turner	Imperial College Healthcare NHS Trust, Charing Cross Hospital (CXH), London UK	Principal Investigator CXH	Richard.turner17@nhs.net
Dr Andrew Innes	Imperial College Healthcare NHS Trust, Hammersmith Hospital, London UK	Co-Investigator	a.innes@imperial.ac.uk
Prof Dragana Milojkovic	Imperial College Healthcare NHS Trust, Hammersmith Hospital, London UK	Co-Investigator	d.milojkovic@imperial.ac.uk
Dr Lucy Cook	Imperial College Healthcare NHS Trust, Hammersmith Hospital, London UK	Co-Investigator	l.cook@imperial.ac.uk
Dr Taryn Youngstein	Imperial College Healthcare NHS Trust, Hammersmith Hospital, London UK	Co-Investigator	t.youngstein@imperial.ac.uk
Prof Mark Thursz	Imperial College London, St Mary's Campus, London UK	Co-Investigator	m.thursz@imperial.ac.uk

Prof Graham Cooke	Imperial College London, St Mary's Campus, London UK	Co-Investigator	g.cooke@imperial.ac.uk
Prof Onn Min Kon	National Heart & Lung Institute, St Mary's Campus, London UK	Co-Investigator	onn.kon@imperial.ac.uk
Prof James Wason	Newcastle University, Newcastle upon Tyne UK	Statistician	james.wason@ncl.ac.uk
Dr Ashley Whittington	London Northwest University Healthcare NHS Trust, Northwick Park Hospital, London.	Principle Investigator	a.whittington@nhs.net
Dr Victoria Parris	London Northwest University Healthcare NHS Trust, Ealing Hospital, London.	Principle Investigator	victoria.parris@nhs.net
Dr Pratap Neelakantan	Royal Berkshire NHS Foundation Trust, Royal Berkshire Hospital, Reading.	Principle Investigator	pratap.neelakantan@royalberkshire.nhs.uk

TRIAL MANAGEMENT GROUP

Name	Affiliation	Role	Contact details
Dr Nichola Cooper	Imperial College Healthcare NHS Trust, Hammersmith Hospital, London UK	Chief Investigator	n.cooper@imperial.ac.uk<br>Tel: 07766546432
Dr Nikhil Vergis	Imperial College Healthcare NHS Trust, St Mary's Hospital (SMH), London UK	Principal Investigator SMH	n.vergis@imperial.uk
Dr Victoria Cornelius	Imperial College London, White City Campus, London UK	Statistician	v.cornelius@imperial.ac.uk
Dr Rachel Phillips	Imperial College London, White City Campus, London UK	Statistician	r.phillips@imperial.ac.uk
Melanie Almonte	Imperial College Healthcare NHS Trust, Hammersmith Hospital, London UK	Research Team Lead	melanie.almonte@nhs.net
Tina Shrutova	Imperial College Healthcare NHS Trust, Hammersmith Hospital, London UK	Senior Research Finance and Data Manager	t.shrutova@nhs.net
Donna Copeland	Imperial College Healthcare NHS Trust, London UK	Divisional Research Manager	donna.copeland@nhs.net
Dr Clio Pillay	Imperial College Healthcare NHS Trust, Hammersmith Hospital, London UK	Trial Manager	clio.pillay@nhs.net

TRIAL STEERING COMMITTEE

Name	Affiliation	Role	Contact details
Dr Nichola Cooper	Imperial College Healthcare NHS Trust, Hammersmith Hospital, London UK	Chief Investigator	n.cooper@imperial.ac.uk<br>Tel: 07766546432
Dr Pratima Chowdary	Royal Free Hospital, London UK	Chair	p.chowdary@nhs.net
Dr Fred Tam	Imperial College London, London UK	Non-independent member	f.tam@imperial.ac.uk
Dr Sarah Brown	Leeds University, Leeds UK	Independent member, Statistician	medsbro@leeds.ac.uk

DATA MONITORING COMMITTEE

Name	Affiliation	Role	Contact details
Dr James Galloway	King's College, London UK	Chair	James.galloway@nhs.net
Dr Arian Laurence	University College London Hospital, London UK	Independent Member	arian.laurence@nhs.net
Sam Norton	King's College, London UK	Independent member, Statistician	sam.norton@kcl.ac.uk

STUDY COORDINATION CENTRE

For general queries, supply of trial documentation, and collection of data, please contact:

Trial Manager: Clio Pillay

Address: Ground Floor, Gary Weston Centre, Hammersmith Hospital, Du Cane Road, W12 0HS

Tel: 0203313 4306 / 077 7855 2277

Fax: n/a

Email:clio.pillay@nhs.net

Web address: TBD

RANDOMISATION

Sealed Envelope

Email:contact@sealedenvelope.com

Web address: https://www.sealedenvelope.com/help/access/access/

CLINICAL QUERIES

Clinical queries should be directed to **Dr Nichola Cooper** or **Dr Nikhil Vergis** who will direct the query to the appropriate person.

SPONSOR

Imperial College London is the main research Sponsor for this study. For further information regarding the sponsorship conditions, please contact the **Head of Regulatory Compliance** at:

Joint Research Compliance Office

Imperial College London and Imperial College Healthcare NHS Trust

Room 215, Level 2, Medical School Building

Norfolk Place

London, W2 1PG

Tel: 0207 594 9480

http://www3.imperial.ac.uk/clinicalresearchgovernanceoffice

This protocol describes the MATIS study and provides information about procedures for entering participants. The protocol should not be used as a guide for the treatment of other participants; every care was taken in its drafting, but corrections or amendments may be necessary. These will be circulated to investigators in the study, but centres entering participants for the first time are advised to contact the trials centre to confirm they have the most recent version. Problems relating to this trial should be referred, in the first instance, to the study coordination centre. This trial will adhere to the principles outlined in the Medicines for Human Use (Clinical Trials) Regulations 2004 (SI 2004/1031), amended regulations (SI 2006/1928) and the International Conference on Harmonisation Good Clinical Practice (ICH GCP) guidelines. It will be conducted in compliance with the protocol, the Data Protection Act and other regulatory requirements as appropriate.

AMENDMENTS

The following amendments and/or administrative changes have been made to this protocol since the implementation of the first approved version:

Amendment Number	Date of Amendment	Protocol Version Number	Type of Amendment	Summary of Amendment
1	14.08.2020	1.8	Substantial Amendment	The opening site will be Imperial College Healthcare NHS Trust in the United Kingdom. Additional UK based sites for the trial will include Hillingdon, Royal Free Hospital, Northwick Park and Chelsea and Westminster added according to trial progress.171 (57 per arm) patients with COVID-19 pneumonia will be recruited to Stage 1, if the trial progresses to Stage 2 an additional maximum of 285 (95 per arm) will be recruited, resulting in a potential sample size of 456 if three trial arms continue for the whole trial (152 per arm). These numbers have been chosen to provide a power of 90% with a maximum 5% chance of an intervention arm being recommended when it provides no improvement over control (5% one-sided family-wise error rate).
2	29.10.2020	1.9	Substantial Amendment	Additional UK sites Leeds and Leicester. Inclusion criteria: CRP now less than or equal to 30 mg/L. Exclusion criteria: removed end stage renal failure, clarified patients on home NIV/CPAP now eligible. Dialysis patients randomised to Ruxolitinib (RUX), will receive only 20mg on dialysis days Week 1, then 10mg on dialysis days Week 2. Pharmacokinetics will be assessed at Baseline, before each dialysis and Day 28. RUX dose for these patients will be assessed after 10 dialysis patients complete Day 14. Research samples will be processed and analysed at Immunology of Infection lab at Imperial College

				London, St Mary's Campus. Added location of research lab. Updated PIS. Updated early discharge. Trial Team and oversight committees updated. Updated unblinding.
3	11.02.2021	2.0	Substantial Amendment	Under Serious Adverse Events, added section 8.3 on Severity Grading. Under Schedule of Events, specified labs to be taken at baseline, Days 1, 7, 14 and 28: Coagulation, Chemistry, Ferritin, Troponin, Procalcitonin; added abbreviation for full blood count (FBC); changed follow-up visit windows from +/-1 day to +/-3 days for Days 7, 14 and 28; changed to +1 day for Day 1 and added -2 days for Day 0. Added Ealing Hospital as a site. Minor document changes: date of last amendment added, clarified abbreviations for ADL and SAE, updated table of contents to reflect addition of 8.3 SAE grading. Addition of research samples to be taken on day 14 and patient information sheet updated to reflect this change. Specification of who can take consent to any competent health care professional as delegated by the CI including doctor, nurse or research practitioner.

ABBREVIATIONS

ADL	Activities of daily living
AE	Adverse Event
ALT	Alanine Aminotransferase
ANC	Absolute Neutrophil Count
ARDS	Adult Respiratory Distress Syndrome
AST	Aspartate Aminotransferase
βhCG	β human chorionic gonadotropin
BP	Blood Pressure
CCO	Central Coordinating Office
CLR	C-type lectin receptors
CPAP	Continuous positive airway pressure
CRF	Case Report Form
CRP	C-Reactive Protein
CXR	Chest X-Ray
DNA	Deoxyribonucleic Acid

FOS	Fostamatinib
ECMO	Extracoporeal membrane oxygenation
EDTA	Ethylene Diamine Tetra-acetic Acid
EoS	End of Study
eGFR	Estimated Glomerula Filtration Rate
FBC	Full Blood Count
FcR	Fc receptors
GI	Gastrointestinal
Hb	Haemoglobin
ICH GCP	International Conference on Harmonisation of Good Clinical Practice
ICL	Imperial College London
ITT	Intention to treat
JAK	Janus kinase
LCC	Local Coordinating Centre
LDH	Lactate dehydrogenase
LFTs	Liver Function Tests
LC	Lymphocyte Count
MSU	Mid-Stream Urine
PCR	Polymerase chain reaction
PI	Principal Investigator
PIS-ICF	Patient Information Sheet-Informed Consent Forms
PT	Prothrombin time
QA	Quality assurance
QC	Quality control
R&D	Research and Development
RNA	Ribonucleic acid
RR	Risk Ratio
RRT	Renal Replacement Therapy
RUX	Ruxolitinib
SAE	Serious Adverse Event
SAR	Severe Adverse Reaction
SOC	Standard of Care
STAT	Signal transducer and activator of transcription proteins
TMG	Trial Management Group
TSC	Trial Steering Committee
USS	Ultrasound Scan
WCC	White Cell Count
WHO	World Health Organisation

TABLE OF CONTENTS

1 Introduction 14
1.1 Background 14
1.2 Purpose of the study 16
1.3 Rationale for the study population 16
2 Study objectives 16
2.1 Primary objective 16
2.2 Secondary Objectives 17
3 Study Design 18
3.1 Sample size 19
3.2 Study sites 19
3.3 Study endpoints 19
4 Statistics and data analysis 20
4.1 Data analysis 20
4.2 Statistical analysis plan 21
4.3 Data management and retention 23
5 Participant entry 24
5.1 Patient selection 24
5.1.1 Inclusion criteria 24
Exclusion criteria 25
5.1.2 25
6 Randomisation and enrolment 25
6.1 Enrolment Procedures forPatients 25
6.2 Randomisation 25
6.3 Unblinding 26
6.4 Case Report Form and PatientNumbers 26
6.5 Specimens and Laboratory handling 26
7 Treatment 26
7.1 Interim analysis to guide dosing regimen 27
7.2 Future changes to Standard of Care 27
7.3 Rationale for Fostamatinib 27
7.3.1 Administration of Fostamatinib 28

Dose modifications for Fostamatinib 28
7.3.2 28
7.4 Rationale for Ruxolitinib 30
7.4.1 Dose modifications for Ruxolitinib 30
7.5 Treatment recommendations for other adverse events 32
7.6 Treatment compliance 32
7.7 Best supportive therapy 32
7.8 Permitted concomitant therapy requiring caution and/or action 32
7.8.1 Strong CYP3A4 inhibitors or dual CYP3A4/CYP2C9 inhibitors 32
7.9 Prohibited medication 33
7.10 Concomitant medication 33
7.11 Supply of study treatment 33
7.12 Treatment duration 34
8 Pharmacovigilance 34
8.1 Definitions 34
8.2 Causality 35
8.3 SAE Grading 36
8.4 Reporting procedures 36
8.5 COVID-19 considerations 37
9 Assessment and Follow-up 40
9.1 Collecting follow-up information 40
9.2 Duration and mode of follow-up 40
9.1 Incidental findings 42
9.2 Lost to follow-up 42
9.3 End of trial 42
10 Trial management 42
10.1 Trial Management Group 42
10.2 Trial Steering Committee (TSC) 42
10.3 Data Monitoring Committee 43
11 Monitoring 43
11.1 Risks and benefits 43
11.2 Monitoring at study coordination centre 44

11.3 Monitoring at local sites 44
11.4 Data and Safety Monitoring 44
12 Ethical considerations and Regulatory Compliance 45
12.1 CTA..... 45
12.2 Ethics approval 45
12.3 Consent..... 45
12.4 Withdrawal of Consent..... 46
12.5 Confidentiality 46
12.6 Indemnity..... 47
12.7 Sponsor..... 47
12.8 Funding 47
12.9 Audits and inspections..... 47
13 Publications and reports..... 47
14 References 48

TABLE OF FIGURES

Figure 1. Classification of COVID-19 Disease States and Potential Therapeutic Targets 15
Figure 2. Schematic of study design 18
Figure 3. Safety Reporting Overview 39

TABLE OF TABLES

Table 1. Modified WHO COVID-19 Severity Scale, from the WHO R&D Blueprint 17
Table 2. Ruxolitinib (RUX) 26
Table 3. Fostamatinib (FOS)..... 27
Table 4. Dose Adjustments for Fostamatinib 28
Table 5. Management of decline in haematologic parameters 31
Table 6. Defining causality..... 35
Table 7. Schedule of events..... 41

SYNOPSIS

TITLE	Randomised multi-arm trial of ruxolitinib (RUX) and fostamatinib (FOS) for COVID-19 pneumonia
AIM	To evaluate efficacy of RUX and FOS compared to standard of care (SOC)in the treatment of COVID-19 pneumonia
PRIMARY OBJECTIVE	To determine the efficacy of RUX and FOS compared to standard of care (SOC)to reduce the proportion of hospitalised patients progressing from mild or moderate to severe COVID-19 pneumonia
SECONDARY OBJECTIVES	• Determine the efficacy of RUX or FOS to reduce mortality<br>• Determine the efficacy of RUX or FOS to reduce the need for invasive ventilation or ECMO<br>• Determine the efficacy of RUX or FOS to reduce the need for non-invasive ventilation<br>• Determine the efficacy of RUX or FOS to reduce the proportion of patients suffering significant oxygen desaturation Determine the efficacy of RUX or FOS to reduce the need for renal replacement therapy<br>• Determine the efficacy of RUX and FOS to reduce the incidence of venous thromboembolism COVID-19 pneumonia<br>• Determine the efficacy of RUX and FOS to reduce the severity onCOVID-19 pneumonia [graded by a modified WHO Ordinal Scale]<br>• Determine the efficacy of RUX or FOS to reduce the level of inflammatory biomarkers<br>• Determine the efficacy of RUX or FOS to reduce the level of serum creatinine<br>• Determine the efficacy of RUX or FOS to reduce duration of hospital admission<br>• Evaluate the safety of RUX and FOS for COVID-19 pneumonia
DESIGN	Multi-site, Two stage, open label, randomized (1:1:1) controlled trial
STUDY DURATION	Treatment is for 14day from baseline. Patients will receive follow-up assessment at 7, 14 and 28 days after the first study dose.<br>Screen Period: up to 7 days<br>Treatment Period: 14 days<br>Follow-up period: Day 14 to 28
SAMPLE SIZE	171 in Stage 1 (57 per arm) and up to an additional 285 in Stage 2 (additional 95 per included arm), to allow for 5% dropout. Patients with mild or moderateCOVID-19 pneumonia will be recruited. Patients will be enrolled into RUX, FOS or SOC groups, as shown in the flow chart.
STUDY SITES	The opening site will be Imperial College Healthcare NHS Trust in the United Kingdom. Additional UK based sites for the trial will include Reading, Hillingdon, Royal Free Hospital, Northwick Park, Chelsea and Westminster, Kingston, and Manchester Royal Infirmary, Leeds, Leicester and Ealing Hospital.
INCLUSION CRITERIA	• Patients age ≥ 18 years at screening<br>• Patients with mild or moderate C19 pneumonia, defined as Grade 3 or 4 severity by the WHO COVID-19 Ordinal Scale

	• Patients meeting criteria:<br>○ Hospitalization *AND*<br>○ SARS-CoV2 infection (clinically suspected* or laboratory confirmed) *AND*<br>○ Radiological change consistent with COVID-19 disease<br>• CRP ≥30mg/L at any time point<br>• Informed consent from patient or personal or professional representative<br>• Agreement to abstain from sexual intercourse or use contraception that is >99% effective for all participants of childbearing potential for 42 days after the last dose of study drug. For male participants, agreement to abstain from sperm donation for 42 days after the last dose of study drug.<br>• Non-English speakers will be able to join the study. If patients are unable to understand verbal or written information in English – hospital translation services will be requested at the participating site for the participant where possible.<br>• Patients normally on non-invasive ventilation such as continuous positive airway pressure (CPAP) at home are eligible
EXCLUSION CRITERIA	• Requiring either invasive or non-invasive ventilation including CPAP or high flow nasal oxygen at any point after hospital admission and before baseline not due to a pre-existing condition (e.g. obstructive sleep apnoea)<br>• Grade ≥5 severity on the modified WHO COVID-19 Ordinal Scale, viz. O_2 saturation < 90% on ≥ 60% inspired oxygen at baseline; non-invasive ventilation; or invasive mechanical ventilation<br>• In the opinion of the investigator, progression to death is inevitable within the next 24 hours, irrespective of the provision of therapy<br>• Known severe allergic reactions to the investigational agents<br>• Child Pugh B or C grade hepatic dysfunction<br>• Use of drugs within the preceding 14 days that are known to interact with any study treatment (FOS or RUX), as listed in the Summary of Product Characteristics<br>• Pregnant or breast feeding<br>• Any medical condition or concomitant medication that in the opinion of the investigator would compromise subjects' safety or compliance with study procedures.<br>• Any medical condition which in the opinion of the principal investigator would compromise the scientific integrity of the study
MAIN STUDY PROCEDURES	Specific assessments occur at:<br>Day 14: death, ventilation status, oxygenation status, renal status<br>Day 28: death, ventilation status, oxygenation status, renal status

PRIMARY ENDPOINTS	• Pairwise comparison of the proportion of patients diagnosed with severe COVID-19 pneumonia within 14 days.<br>• Severe COVID-19 pneumonia is defined by a modified WHO COVID-19 Ordinal Score ≥ 5, comprising the following indicators of disease severity:<br>o Death *OR*<br>o Requirement for invasive ventilation *OR*<br>o Requirement for non-invasive ventilation including CPAP or high flow oxygen *OR*<br>o O_2 saturation < 90% on ≥60% inspired oxygen
SECONDARY ENDPOINTS	• Pairwise comparison of the proportion, median or odds ratio of each of the parameters below at 14 and 28 days between RUX or FOS versus SOC:<br>o Mortality<br>o Invasive ventilation or ECMO<br>o Non-invasive ventilation including CPAP or high flow nasal oxygen<br>o Renal replacement therapy<br>o Venous thromboembolism<br>Length of stay<br>o Serious adverse events and discontinuations of study arms<br>o Inflammatory markers<br>o Change in pneumonia severity on the modified WHO COVID-19 Ordinal Scale
EXPLORATORY ENDPOINTS	• Nasosorption biomarkers at baseline, Day 14, Day 28<br>• Biomarkers of disease including plasma, PBMC and DNA changes<br>Further secondary and exploratory objectives are not the focus of this study but maybe adopted using routine healthcare records (e.g. NHS Digital or equivalent international databases) and relevant research studies (e.g. UK Biobank or equivalent international resources). This will allow subsidiary analyses of the effect of the study treatments on particular non-fatal events (e.g. ascertained through linkage to Hospital Episode Statistics), the influence of pre-existing major co-morbidity (e.g. chronic heart disease and use of immunosuppressive drugs) and longer-term outcomes (e.g. 6 month survival) as well as in particular sub-categories of patient (e.g. by genotype).

1 INTRODUCTION

1.1 BACKGROUND

COVID-19 pneumonia is characterised by respiratory and multi-organ failure in the context of marked systemic inflammation. It is caused by Severe AcuteRespiratory Syndrome Coronavirus 2 (SARSCoV2) infection. The

hallmark of severe disease is hypoxia and a radiological pattern of acute lung injury that shares features with Acute Respiratory Distress Syndrome (ARDS). Early features of COVID-19 result from host viral response and typically include symptoms such as fever and dry cough. Later features, typically occurring beyond 7 days, are characterised by marked and progressive systemic inflammation, identified by elevations in a plethora of inflammatory molecules such as C-reactive protein, ferritin and IL6. In a subset of patients, hyperinflammatory responses drive acute lung injury and may result in catastrophic multi-organ failure and death(Huang et al., 2020).

Figure 1. Classification of COVID-19 Disease States and Potential Therapeutic Targets

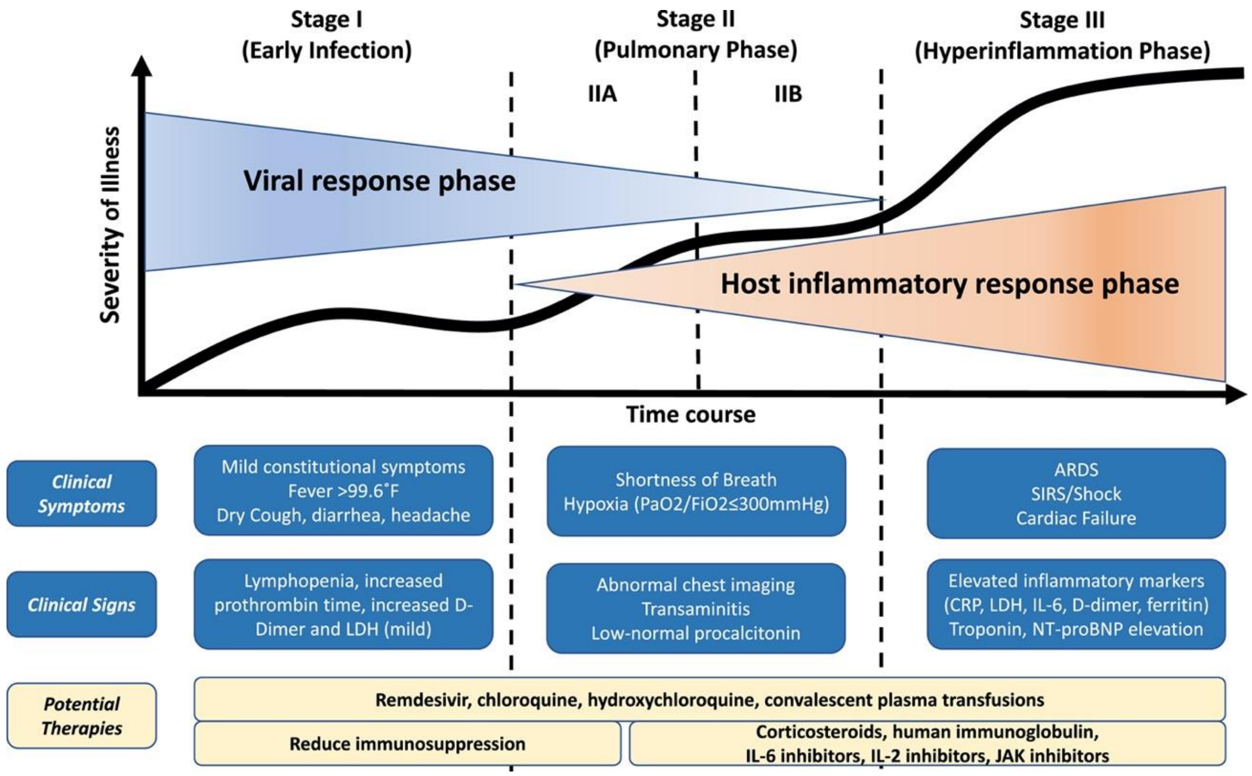

(Siddiqi & Mehra, 2020)

The aetiology of COVID-19 induced ARDS is incompletely understood but appears to be associated with lung inflammation effected by a monocytic and neutrophilic infiltration, elevated cytokine levels and tissue damage (Siddiqi & Mehra, 2020). Elevations in circulating inflammatory molecules are associated with poor prognosis. In particular, the COVID-19 hyperinflammatory response syndrome is associated thrombotic complications which are postulated to drive cardiac dysfunction and microvascular thrombi, suggested by elevations in troponin and D-dimer, respectively (Ruan et al., 2020). Similar hyperinflammatory responses are also seen in macrophage

activation syndromes such as haemophagocytic lymphohistiocytosis, or in the cytokine release syndrome associated with chimeric antigen receptor T cell therapy (Singanayagam et al., 2018). Further, preliminary data from China and Italy have shown immediate resolution of symptoms using anti-IL6 therapy and JAK/STAT inhibitors in patients with severe disease (Richardson et al., 2020; Wu & Yang, 2020). There may be an early window of opportunity to treat the COVID-19 hyperinflammatory syndrome before acute lung injury leads to organ failure. There are currently no approved treatments for covid-19 pneumonia. This is a protocol for a randomised controlled, multi-arm trial of early intervention with inflammatory signal inhibitors.

1.2 PURPOSE OF THE STUDY

A number of therapeutic interventions targeting inflammatory signalling might reduce the severity of the inflammatory response phase resulting in amelioration of the lung damage thereby averting respiratory failure and the need for mechanical ventilation. This trial aims to evaluate the efficacy of two inhibitors of key signalling pathways using drugs which are already licensed for use in other clinical indications.

1.3 RATIONALE FOR THE STUDY POPULATION

Patients with COVID-19 infection in the community may be asymptomatic or experience only mild transient symptoms which do not merit experimental therapeutic intervention. Patients who are admitted to hospital due to the severity of their symptoms are invariably in the inflammatory phase of the disease with dyspnoea and/or hypoxia indicating involvement of the lower respiratory tract. Data from our own hospital as well as Chinese and Italian experiences indicate that around 30% of hospitalised patients will deteriorate during their admission resulting in the need for ventilatory support. The study population is therefore selected to evaluate whether the experimental treatments are effective in prevention of clinical deterioration in a hospitalised population.

2 STUDY OBJECTIVES

2.1 PRIMARY OBJECTIVE

The primary objective is to determine the efficacy of RUX and FOS to reduce the proportion of hospitalised patients progressing from mild/moderate to severe COVID-19 pneumonia.A modified WHO COVID-19 Severity Ordinal Scale (COVID-19 Therapeutic Trial Synopsis published 18th February 2020) will be used to grade clinical deterioration from Hospitalised Mild Disease (<5) to Hospitalised Severe Disease (≥ 5). The modification includes an additional grade for Hospitalised Severe Disease that allows the capture of clinical deterioration in patients for whom escalation in organ support is not offered. Patients are eligible for recruitment to MATIS at grades 3 or

4. These patients stand to gain the greatest benefit from inflammatory signal inhibitors that may ameliorate the cytokine storm and prevent organ failure.

Table 1. Modified WHO COVID-19 Severity Scale, from the WHO R&D Blueprint

Patient state	***Descriptor***	***Grade***
Uninfected	No clinical or virological evidence of infection	0
Ambulatory	No limitation of activities	1
	Limitation of activities	2
Hospitalised mild disease	Hospitalised, no oxygen therapy	3
	Oxygen by mask or nasal prongs	4
Hospitalised severe disease	$SpO_2 < 90\%$ on $FiO_2 \geq 60\%$ by face mask	5
	Non-invasive ventilation, CPAP or high-flow oxygen	6
	Intubation and mechanical ventilation	7
	Ventilation + additional organ support (vasopressors, RRT, ECMO)	8
Dead	Death	9

Published in the COVID Therapeutic Trial Synopsis, February 18th 2020

2.2 SECONDARY OBJECTIVES

- Determine the efficacy of RUX or FOS to reduce mortality
- Determine the efficacy of RUX or FOS to reduce the need for invasive ventilation and/or ECMO
- Determine the efficacy of RUX or FOS to reduce the need for non-invasive ventilation including CPAP or high flow nasal oxygen
- Determine the efficacy of RUX or FOS to reduce the proportion of patients suffering clinically significant oxygen desaturation
- Determine the efficacy of RUX or FOS to reduce the need for renal replacement therapy
- Determine the efficacy of RUX and FOS to reduce the incidence of venous thromboembolism COVID-19 pneumonia
- Determine the efficacy of RUX and FOS to improve the severity of COVID19 pneumonia on a modified WHO COVID19 Ordinal Scale
- Determine the efficacy of RUX or FOS to reduce blood ferritin, CRP, LDH and D-dimer

- Determine the efficacy of RUX or FOS to reduce the level of inflammatory biomarkers
- Determine the efficacy of RUX or FOS to reduce the level of serum creatinine
- Determine the efficacy of RUX or FOS to reduce duration of hospital admission
- Evaluate the safety of RUX and FOS for COVID19 pneumonia

3 STUDY DESIGN

This is a multi-site, two stage, open label, randomized (1:1:1) controlled trial.

Figure 2. Schematic of study design

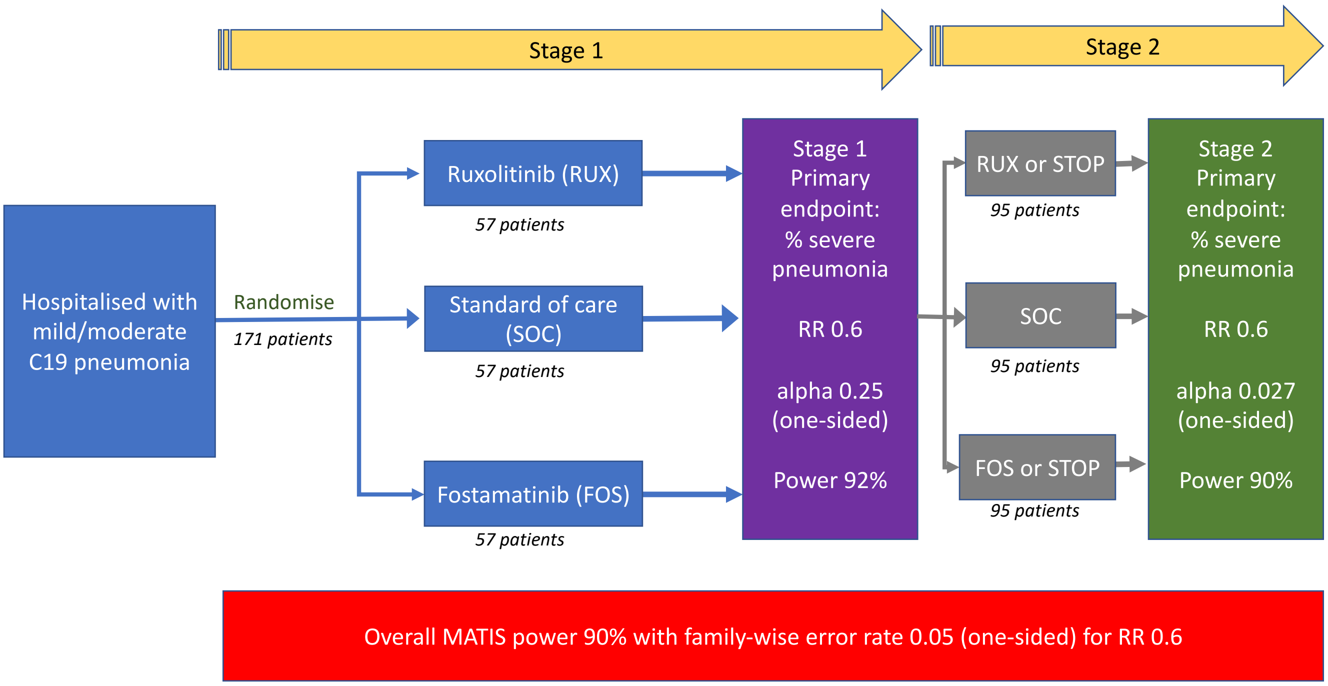

Treatment is for 14 days from baseline. Patients will receive follow-up assessment at 7, 14 and 28 days after the first dose.

The time allowed for screening assessments allows time for eligibility assessments to be included.

- Screening Period: up to 7 days to the day randomisation
- Treatment Period: 14 days
- Follow-up period: Day 14 to 28

Patients who have recovered and are fit for discharged from hospital during the treatment period, will be discharged home with trial medication to complete a fixed 14 day course. Such patients will be followed up weekly with telephone monitoring until day 28 and additional blood test monitoring where practically possible.

3.1 SAMPLE SIZE

171 (57 per arm) patients with COVID-19 pneumonia will be recruited to Stage 1, if the trial progresses to Stage 2 an additional maximum of 285 (95 per arm) will be recruited, resulting in a potential sample size of 456 if three trial arms continue for the whole trial (152 per arm). These numbers have been chosen to provide a power of 90% with a maximum 5% chance of an intervention arm being recommended when it provides no improvement over control (5% one-sided family-wise error rate).

Patients will be enrolled into RUX, FOS or SOC groups, as shown in the flow chart.

3.2 STUDY SITES

The trial will commence at one site, Imperial College Healthcare NHS Trust in the UK, with further UK based sites that will include Reading, Hillingdon, Royal Free Hospital, Northwick Park, Chelsea and Westminster, Kingston, Manchester Royal Infirmary, Leeds, Leicester and Ealing Hospital.

3.3 STUDY ENDPOINTS

Primary endpoints

The primary endpoint is progression from mild to severe COVID-19 pneumonia within 14 days in hospitalised patients. Patients are recruited at a WHO COVID-19 Severity Score of 3 and 4 and the primary endpoint is the comparison of patients whose COVID-19 pneumonia progresses to a severity score ≥ 5 on the modified WHO Ordinal Scale. Specifically, the primary endpoint is met when the following are recorded within 14 days:

- Death
- Requirement for invasive ventilation
- Requirement for non-invasive ventilation including CPAP and high flow nasal oxygen
- O_2 saturation < 90% on $\geq$60% inspired oxygen

Secondary endpoints

Comparison of the incidence, time-to-event, median or odds ratio of each of the parameters below at 14 and 28 days between RUX or FOS versus SOC:

- Mortality
- Invasive ventilation
- Non-invasive ventilation including CPAP and high flow nasal oxygen
- Renal replacement therapy

- Venous thromboembolism
- Inflammatory markers CRP, LDH, ferritin, D-dimer
- Serum creatinine
- Length of stay
- SSARs and discontinuations of study arms
- Change in severity on the modified WHO COVID-19 Ordinal Scale

Further secondary and exploratory objectives are not the focus of this study but maybe adopted using routine healthcare records (e.g. NHS Digital) and relevant research studies (e.g. UK Biobank and Genomics England). This will allow subsidiary analyses of the effect of the study treatments on particular non-fatal events (e.g. ascertained through linkage to Hospital Episode Statistics), the influence of pre-existing major co-morbidity (e.g. chronic heart, lung, kidney, liver disease, diabetes and use of immunosuppressive drugs) and longer-term outcomes (e.g. 6 month survival) as well as in particular sub-categories of patient (e.g. by genotype).

Exploratory endpoints

Blood samples will be taken as per protocol schedule to evaluate changes in immune cells and platelets over time to look for biomarkers to predict response. This will include fresh blood analysis, and collecting DNA, plasma, serum and PBMCs for freezing and future analysis. Samples will be stored under Imperial College Tissue Bank.

4 STATISTICS AND DATA ANALYSIS

4.1 DATA ANALYSIS

The aim of the trial (stage 1 and 2) is to test whether RUX and FOS (separately) provide significant improvement in primary outcome compared to SOC. To do this we will test the two null hypotheses and provide estimated treatment effect (Ors) with 95% confidence intervals (Cis).

Interim analysis (Stage 1)

The aim of the Stage 1 interim analysis is to provide an opportunity to stop enrolling patients to an intervention arm that is not showing sufficient promise of efficacy. The Stage 1 review will be undertaken by a statistician that is independent of ICTU and overseen by the data monitoring committee who will advise on whether the trial should continue with both intervention arms, with one intervention arm, or stop at this stage. This decision

will be based on a suggested one-sided p<0.25 although interpretation of the p-value will be on the continuous scale as strength against the null hypotheses and will not be restricted to a single binary threshold. Our sample size calculations for both stages ensure we have a good prospect of these achieving these aims.
Current rate of severe pneumonia in the standard of care is 50%. In the case that an experimental arm reduces this to 30% (relative risk, RR, 0.6), then the design provides a 92% chance (one sided alpha 0.25) of it being recommended to continue to stage 2.

Full trial (Stage 2)
If the trial is recommended to continue at Stage 2 based on DMC interim review the full trial will need to recruit an additional 95 for each arm that continues (152 per arm in total; 456 to the complete trial). This will provide 90% power to detect a change of RR of 0.6 (50% to 30%), with one-sided alpha of 0.027 (5% FWER) and has been inflated for 5% for missing outcome data. Here the power is the chance of an effective treatment passing stage 1 and being recommended after stage 2. The design recommends stopping an intervention arm at stage 1 if the one-sided p-value is >0.25. The above power assumes this rule is always followed and the FWER assumes it is not followed (hence the actual FWER will be lower if the rule is always followed).

4.2 STATISTICAL ANALYSIS PLAN

Analysis will be on an intention to treat (ITT) population including all randomized patients, multiple imputation will be used to include participants who have missing outcome data. Comparisons for efficacy will be made between all participants randomised to the different treatment arms, irrespective of whether they received their allocated treatment ("ITT" analyses). Comparisons for safety will be made between all participants randomised to the different treatment arms who have received at least one dose of study medication. Multiple imputation makes a missing at random assumption and a sensitivity analysis around the assumptions made will be performed using controlled multiple imputation that permits exploration of a not missing at random assumption. A complete case analysis will also be performed. The primary outcome will be analysed as binary with participants categorised WHO COVID-19 Ordinal Score $<5/\geq 5$ by 14 days.

For Stage 1 analysis (after a target recruitment of 171 participants) we will calculate the Wald test statistics from separate logistic regression modelsfor RUX vs SOC and FOS vs SOC, adjusted for stratification variables, with 95% confidence intervals and p-values. P-values will be one-sided and judged at the 0.25 level.

If at least one intervention arm shows promise, then stage 2 will recruit a further 95 per arm (the maximum combined sample size of stages1 and 2is 456 patients). The stage 2 analysis will use a one-sided p-value threshold of 0.027. This has been chosen to control the maximum chance of recommending an ineffective treatment at 0.05.

Contamination is a potential threat in many C19 trials, as standard of care is continuously evolving, and co-enrolment to ongoing trials present a real challenge if unequal enrolment occurs across arms. For participants who were co-enrolled to a trial at the same time, or prior to the randomisation to this trial, we will adjust the final analysis though covariate adjustment. For participants who were subsequently co-enrolled after randomisation, we treat this as a post-randomisation variable and undertake a supplementary analysis to adjust for this variable along with the receipt of post-randomisation rescue medication.

In Stage 2 a logistic regression model will be used to assess the treatment effect of RUX and FOS compared to SOC. The model will include the treatment arms, baseline modified WHO Severity Score, trial co-enrolment at baseline, and randomisation stratification variables viz. site and age (<65 vs ≥65). As well as reporting OR and 95% Cis, the model will be used to estimate the difference in proportion of participants with modified WHO COVID-19 Ordinal Score ≥5. The treatment effect in the absence of post-randomisation co-enrolment and receipt of rescue medication will be estimated in a supplementary analysis where participants' outcomes after either will be treated as missing and a controlled multiple imputation using a delta based approach will be used.

For the primary outcome, progression to severe COVID-19 disease within 14 days, discharge alive from the admitting hospital before 14 days will assume safety from the event (in the absence of additional data confirming otherwise). An additional sensitivity analysis will be performed if there is a change of dose after review of the first thirty patients.

For time-to-event analyses required for secondary endpoints such as time to discharge, each treatment group will be compared with SOC and modelled using a proportional hazards time-to-event model adjusted for randomisation stratification variables. Kaplan-Meier estimates for the time to event will also be plotted (with confidence intervals). The log-rank 'observed minus expected' statistic (and its variance) will be used to estimate the average event rate ratio (and its confidence interval) for those allocated to each treatment group versus the no additional treatment group.

Pre-specified subgroup analysis will be conducted for the primary outcome using the statistical test for interaction (or test for trend where appropriate) for the following: disease severity as graded by the WHO COVID-19 Ordinal Severity Scale; time since onset of symptoms; sex; age; and comorbidities obesity, chronic heart, lung, kidney and liver disease, diabetes, immunocompromised and smoking status. Pre-specified subgroup analyses will also be conducted for the primary outcome using the statistical test for interaction (or test for trend where appropriate) for patients with admission CRP >200mg/L and/or D-dimer 2500ng/mL.

Further details will be fully described in the Statistical Analysis Plan (SAP).

4.3 DATA MANAGEMENT AND RETENTION

Clinical research teams will use the bespoke study web-based applications for study management and to record participant data (including case report forms) in accordance with the protocol. Data will be held in central databases located at the CCO or on secure cloud servers. In some circumstances (e.g. where there is difficulty accessing the internet or necessary IT equipment), paper case report forms may be required with subsequent data entry by either LCC or CCO staff. Although data entry should be mindful of the desire to maintain integrity and audit trails, in the circumstances of this epidemic, the priority is on the timely entry of data that is sufficient to support reliable analysis and interpretation about treatment effects. CCO staff will be responsible for provision of the relevant web-based applications and for generation of data extracts for analyses.
All data access will be controlled by unique usernames and passwords, and any changes to data will require the user to enter their username and password as an electronic signature in accordance with regulatory requirements. Staff will have access restricted to the functionality and data that are appropriate for their role in the study.

Data retention

Data and all appropriate documentation will be securely archived for a minimum of 10 years after the completion of the study, including the follow-up period in accordance with College policy

5 PARTICIPANT ENTRY

5.1 PATIENT SELECTION

In hospital, potential participants will be identified through hospital workers upon presentation at recruiting sites and through public health agencies

5.1.1 INCLUSION CRITERIA

- Patients age ≥ 18 years at screening
- Patients meeting criteria of:
  - Hospitalization *AND*
  - SARS-CoV2 infection (clinically suspected[*] or laboratory confirmed) *AND*
  - Radiological change consistent with COVID-19 disease
- Mild or moderate (Grade 3 or 4 severity by modified WHO COVID-19 Ordinal Scale) C19 pneumonia
- CRP ≥30mg/L at any time point
- Informed consent from patient or personal or professional representative
- Agreement to abstain from sexual intercourse or use contraception that is >99% effective for all participants of childbearing potential for 42 days[†]
- For male participants, agreement to abstain from sperm donation for 42 days after the last dose of study drug[‡]
- Non-English speakers will be able to join the study. If patients are unable to understand verbal or written information in English – hospital translation services will be requested at the participating site for the participant where possible
- Patients normally on non-invasive ventilation such as continuous positive airway pressure (CPAP) at home are eligible

[*] Clinical suspicion of COVID-19 pneumonia will be confirmed by two independent clinicians.
[†] Females must be either post-menopausal for at least 1 year or surgically sterile; or if female of child-bearing potential, must not be pregnant or lactating and must agree to use an acceptable method of birth control throughout the duration of the trial and for 30 days following the last dose. Acceptable methods of birth control are defined as: hormonal contraception (pill, injection or implant) used consistently for at least 30 days prior to enrolment, intrauterine device (IUD), double-barrier (ie, condom and spermicide, or condom and diaphragm), or true abstinence (when this is in line with the preferred and usual lifestyle of the subject).
[‡]Male subject with a partner of childbearing potential must agree to use an appropriate double-barrier method of contraception during the time interval between administration of the first dose of study drug and 30 days following the last dose, or must agree to true abstinence (when this is in line with the preferred and usual lifestyle of the subject).

5.1.2 EXCLUSION CRITERIA

- Requiring either invasive or non-invasive ventilation including CPAP or high flow nasal oxygen at any point after hospital admission and before baseline, not related to a pre-existing condition (e.g. obstructive sleep apnoea)
- Grade ≥5 severity on the WHO COVID-19 Ordinal Scale
- O_2 saturation < 90% on ≥ 60% inspired oxygen at baseline
- In the opinion of the investigator, progression to death is inevitable within the next 24 hours, irrespective of the provision of therapy
- Any medical condition or concomitant medication that in the opinion of the investigator would compromise subjects' safety or compliance with study procedures.
- Any medical condition which in the opinion of the principal investigator would compromise the scientific integrity of the study
- Known severe allergic reactions to the investigational agents
- Use of drugs within the preceding 14 days that are known to interact with any study treatment (FOS or RUX), as listed in the Summary of Product Characteristics
- Child Pugh B or C grade hepatic dysfunction
- Pregnant or breast feeding

6 RANDOMISATION AND ENROLMENT

6.1 ENROLMENT PROCEDURES FORPATIENTS

Eligible patients who have given informed consent to participate will be enrolled to the study. With due consideration to the circumstances of possible admission to a high-level isolation unit a full study information sheet will be given subsequent to the consent discussion. All patients will have clinical information collected either directly through examination including a review of medical, contact and travel history, or from available medical notes. Information will be recorded in the case report form.

6.2 RANDOMISATION

Patients will be randomized to one of three actively recruiting trial arms, RUX, FOS or SOC. Eligible patients will be allocated using a central web-based randomisation service that uses randomisation sequences with random

block sizes that are stratified by age (<65 vs ≥65) and site. If stage 2 occurs with one intervention arm stopped then the randomisation ratio will be 1:1.

6.3 UNBLINDING

Analysis will be undertaken primarily by the trial statistician who will be blinded to treatment assignment. Any analysis of primary efficacy outcomes or data with the potential to unblind the trial statistician (e.g. adherence to allocated intervention) will be undertaken by an independent statistician from Newcastle University. An independent statistician based in Imperial Clinical Trials Unit will hold the information to unblind the data for closed DMC discussions.

6.4 CASE REPORT FORM AND PATIENTNUMBERS

Case Report Forms (CRFs) will be used to collect data at enrolment to this study. Patient numbers will be assigned a 2-digit site code and a 3-digit patient number sequentially beginning with 001.

6.5 SPECIMENS AND LABORATORY HANDLING

All samples will be analysed at Imperial College Healthcare NHS Trust laboratories (operating as North West London Pathology). Research samples will be processed and analysed at the Immunology of Infection lab at Imperial College London, St Mary's Campus.

7 TREATMENT

It is accepted that SOC may change during a rapidly evolving pandemic. Co-enrolment to other trials and rescue therapy, either pre- or post-randomisation, is permitted and will be accounted for in the statistical analysis.

Table 2. Ruxolitinib (RUX)

Period	***Dose***	***Frequency***
Day 1-7	10mg	Twice daily
Day 8-14	5mg	Twice daily

Table 3. Fostamatinib (FOS)

Period	*Dose*	*Frequency*
Day 1-7	150mg	Twice daily
Day 8-14	100mg	Twice daily

7.1 INTERIM ANALYSIS TO GUIDE DOSING REGIMEN

There is no prior evidence with which to base particular dosing regimens for RUX or FOS for the treatment of COVID-19 pneumonia. After the first 10 patients have been recruited in each arm, an interim analysis will therefore be conducted to test whether a mean 25% decrease in CRP (an inflammatory biomarker) has been achieved in each of the treatment arms compared to SOC at Day 14. This analysis will inform a recommendation by the DMC to consider dose escalation for subsequent patients. In the event of that the dosing regimen is changed for subsequent patients, all patients will be included in the final analysis of the primary outcome, but a sensitivity analysis will be performed that excludes patients treated with the prior dosing.

7.2 FUTURE CHANGES TO STANDARD OF CARE

The study team will remain cognisant of the prevailing global literature during the current pandemic on effective therapies for COVID-19 pneumonia. If a treatment emerges with efficacy and safety profile that is superior to SOC then options for adoption of the new therapy as SOC will be discussed with the DMC. Specifically, the impact that the new SOC might have on original trial statistical parameters and outcomes measures will be considered.

7.3 RATIONALE FOR FOSTAMATINIB

Fostamatinib is a tyrosine kinase inhibitor with activity against spleen tyrosine kinase (SYK). It has approved for the treatment of thrombocytopenia in adult patients with chronic immune thrombocytopenia (ITP). Studies of severe acute respiratory syndrome (SARS), induced by a related coronavirus, suggest that pathogenesis relies on a series of SYK-dependent events. SYK activity mediates cytokine and chemokine release induced by the activation of C-type lectin receptors (CLR) and immunoglobulin Fc receptors (FcR) resulting in neutrophil and monocyte lung ingress, sequential activation of neutrophil extracellular traps and the activation of lung epithelium and multiple myeloid cell. This is followed by inflammation and tissue destruction that contribute to ARDS. Fostamatinib, by inhibiting SYK activity, can block the production and release of cytokines induced via CLR and FcR activation, thus potentially ameliorating the cytokine storm that often precedes ARDS.

Fostamatinib is the only SYK inhibitor approved for clinical use, offering a distinct anti-inflammatory potential with a proven safety profile. Moreover, its anti-inflammatory effect is specific as preclinical toxicity studies showed that SYK inhibition with fostamatinib did not adversely affect innate immune responses in three different host resistance models, which is consistent with its clinical safety profile

7.3.1 ADMINISTRATION OF FOSTAMATINIB

Administration

For subjects who become unable to receive fostamatinib treatment orally (e.g, intubated subjects), tablets can be crushed until granular with an approximate particle size <2 mm (based on the diameter of an NG tube), added to approximately 10 mL of water (or suitable volume for administration through a feeding tube), and stirred to mix before administration through an enteral feeding tube. The tablet is not expected to be fully dissolved prior to administration.

Caveats

The safety and efficacy of fostamatinib was evaluated using intact tablets only, so we do not have specific safety or efficacy data to support crushing tablets and administering through an NG tube. However, we have internal data on file that supports the stability of the crushed tablet for approximately 24 hours in a slightly basic pH (e.g. sterile water).

Crushing tablets

Based on findings from animal studies and the mechanism of action, fostamatinib can cause fetal harm when administered to a pregnant woman. Therefore, it is recommended that appropriate precautions should be followed during preparation of the crushed tablets: the tablets should be crushed and solubilized in water within a hood, and the individual preparing the crushed tablet wear gloves and a particle mask.

7.3.2 DOSE MODIFICATIONS FOR FOSTAMATINIB

Table 4. Dose Adjustments for Fostamatinib

Hypertension	Recommended Action
Stage 1: systolic between 130-139 or diastolic between 80-89 mmHg	- Monitor, or initiate or increase dosage of antihypertensive medication for subjects with increased cardiovascular risk and adjust as needed until BP is controlled, in accordance with localstandards.

Stage 2: systolic at least 140 or diastolic at least 90 mmHg	- Initiate or increase dosage of antihypertensive medication and adjust as needed until BP iscontrolled.
Hypertensive crisis: systolic over 180 and/or diastolic over 120 mmHg	- Interrupt or discontinue study drug.<br>- Initiate or increase dosage of antihypertensive medication and adjust as needed until BP is controlled. If BP returns to less than the target BP, resume study drug at same daily dose.

Toxicity	Recommended Action
AST/ALT is 5 x ULN or higher and total BL is less than 2 x ULN	- Interrupt study drug.<br>- Recheck LFTs every 72hours:<br>- If AST and ALT decrease, recheck until ALT and AST are no longer elevated (below 1.5xULN) and total BL remains less than 2xULN; resume study drug at next lower daily dose.<br>- If AST/ALT persist at 5xULN or higher for 2 weeks or more, discontinue study drug.
AST/ALT is 3 x ULN or higher and less than 5 x ULN	If patient is symptomatic (e.g., nausea, vomiting, abdominal pain):<br>- Interrupt study drug.<br>- Recheck LFTs every 72 hours until ALT/AST values are no longer elevated (below 1.5 x ULN) and total BL remains less than 2 xULN.<br>- Resume study drug at next lower daily dose.<br><br>If patient is asymptomatic:<br>- Recheck LFTs every 72 hours until ALT/AST <1.5 x ULN) and total BL remains less than 2xULN.<br>- Consider interruption or dose reduction of study drug if ALT/AST and TBL remain in this category (AST/ALT is 3 to 5 x ULN; and total BL remains less than 2 xULN).<br>- If interrupted, resume study drug at next lower daily dose when ALT/AST are no longer elevated (<1.5xULN) and total BL remains <2 xULN.<br>- If AST/ALT persist at 5 x ULN or higher for 2 weeks or more, discontinue study drug.
AST/ALT is 3 x ULN or higher and total BL >2 x ULN	- Check direct and indirect BL levels. If direct BL is >2x ULN then discontinue study drug, if only indirect BL is >2x ULN then monitor as above.
Elevated unconjugated (indirect) BL in absence of other LFT abnormalities	- Continue study drug with frequent monitoring since isolated increase in unconjugated (indirect) BL may be due to UGT1A1 inhibition or the underlying disease.

Diarrhoea	- Manage diarrhoea using supportive measures (e.g., dietary changes, hydration and/or antidiarrheal medication) early after the onset until symptom(s) have resolved.<br>- If symptom(s) become severe (Grade 3 or above), temporarily interrupt study drug.<br>- If diarrhoea improves to mild (Grade 1), resume study drug at the next lower daily dose.
Neutropenia	- If absolute neutrophil count decreases (ANC <1.0 x 109/L) (or is low at baseline) give G-CSF support to ensure NP>1.0 x 109/L

7.4 RATIONALE FOR RUXOLITINIB

JAK and STAT molecules are proteins that transduce extracellular stimulation into intracellular signalling, leading to expression of a host of inflammatory cytokines in a variety of immune cells (Elli et al., 2019). Ruxolitinib is a JAK1/JAK2 inhibitor approved for clinical use in the treatment of splenomegaly, myelofibrosis, polycythaemia vera and graft-versus-host disease. It is an oral agent with a rapid mode of action. Inhibition of STAT3 activation occurs within 2 hours of RUX administration and downregulates IL-6 and IL-23 signalling important for the pro-inflammatory effects of Th17 cells. Further, RUX administration leads to in reductions in serum levels TNFa and CRP. In addition, JAK2 inhibitors have been shown to block receptor-mediated endocytosis, thereby preventing viral cellular entry and assembly (Jagasia et al., 2020).

7.4.1 DOSE MODIFICATIONS FOR RUXOLITINIB

End stage renal failure patients

Patients with glomerular filtration rate (GFR)<15 ml/min (dialysis and non-dialysis patients) will receive 20mg three times a week the first week and for the second week 10 mg three times a week (taken at the end of dialysis if on dialysis).

Patients with GFR ≥15 ml/min to < 30 ml/min, 5mg twice a day for 1 week, then 5 mg once a day for 1 week.

For dialysis patients, pharmacokinetic (PK) levels will be assessed before each dialysis and at Day 14 and Day 28. Ruxolitinib dose for these patients will be assessed after 10 dialysis patients complete these doses to Day 14.

Dose reductions

Dose reductions or interruptions for non-haematological toxicity attributed to ruxolitinib are permitted in order to allow the patient to continue on the study. For haematological toxicity, the counts should be supported with blood product support ensuring that the platelet count remains > 50 x 109/L. The dose of ruxolitinib may be

reduced in response to certain toxicities as detailed below. Doses of supportive medications should be adjusted according to standard practice. If absolute neutrophil count decreases (ANC <1.0 x 109/L) (or is low at baseline) give G-CSF support to ensure NP> 1.0 x 109/L

Table 5. Management of decline in haematologic parameters

Parameter(s) at Time of Decline	Action
PLT < 50 x 10^9/L	Transfuse platelets to ensure >50 x 109/L
ANC < 1.0 x 10^9/L	Support with G-CSF to ensure NP>1.0 x 109/L

In response to a decline in renal function (eGFR < 30ml/min) OR hepatic function (AST/ALT > 1.5 x ULN), the dose should be reduced by 50%, to be administered twice daily and patients should be carefully monitored. The dose can be re-escalated if eGFR subsequently increases above 30ml/min OR AST/ALT decreases to < 1.5 x ULN as applicable.

Potential for Ruxolitinib-induced liver injury

Participants with transaminase increase combined with total bilirubin increase may be indicative of potentially severe ruxolitinib-induced liver injury and should be considered as clinically important events and assessed appropriately to establish the diagnosis. The required clinical information, as detailed below, should be sought to obtain the medical diagnosis of the most likely cause of the observed laboratory abnormalities.
The threshold for potential ruxolitinib-induced liver injury may depend on the participant's baseline AST/ALT and total bilirubin value; participants meeting any of the following will require further follow-up:

- For participants with normal ALT and AST and total bilirubin value at baseline: AST or ALT > 3.0 x ULN combined with total bilirubin > 2.0 x ULN
- For participants with elevated AST or ALT or total bilirubin value at baseline: (AST or ALT > 2 x baseline or AST or ALT >300 U/L) whichever occurs first combined with (total bilirubin > 2 x baseline AND > 2.0 x ULN)

Other causes of abnormal liver tests should also be considered, and their role clarified before ruxolitinib is assumed as the cause of liver injury. A detailed history, including relevant information such as review of ethanol consumption, concomitant medications, herbal remedies, supplement consumption, history of any pre-existing

liver conditions or risk factors, should be collected. Laboratory tests to be done include ALT, AST, total bilirubin, direct and indirect bilirubin, GGT, LDH, prothrombin time (PT)/INR, alkaline phosphatase, albumin, and creatine kinase.

7.5 TREATMENT RECOMMENDATIONS FOR OTHER ADVERSE EVENTS

Grade 1 or 2: maintain dose level

Grade 3: Reduce dose level 50% until resolved to ≤ Grade 2

Grade 4: Hold dose and then discontinue from study

7.6 TREATMENT COMPLIANCE

The local trial pharmacist will be responsible for maintaining and updating the drug accountability log in the study pharmacy file, which will be used to monitor compliance. All unfinished packs will be returned to the trial pharmacist who will count and document any unused medication. All IMP can then be destroyed in accordance with local pharmacy practice and this will be documented on the drug destruction log in the hospital pharmacy file.

7.7 BEST SUPPORTIVE THERAPY

All patients will receive best supportive therapy for COVID-19 as per physician's discretion.

7.8 PERMITTED CONCOMITANT THERAPY REQUIRING CAUTION AND/OR ACTION

Patients may receive anti-emetics, calcineurin inhibitors, azole fungal prophylaxis or broad-spectrum antibiotics (either semi-synthetic penicillin or third generation cephalosporin with vancomycin, gentamycin or equivalent). Use of sedatives should be closely monitored for potential drug-drug interaction effects. Use of oral, injected or implanted hormonal methods of contraception are allowed while on ruxolitinib. Ruxolitinib dose adjustments may be required, particularly in patients treated with CYP450 modulators.

7.8.1 STRONG CYP3A4 INHIBITORS OR DUAL CYP3A4/CYP2C9 INHIBITORS

Upon initiation of a strong CYP3A4 inhibitor, including clarithromycin or a dual CYP3A4/CYP2C9 inhibitor including fluconazole up to a dose of 200 mg, suggest change antibiotic to azithromycin or doxycycline. If patient must remain on a strong CYP3A4 inhibitor or dual CYP3A4/CYP2C9 inhibitor, then the dose of ruxolitinib should be reduced (e.g. by 50%) with daily monitoring of haematology parameters and clinical signs and symptoms of ruxolitinib related adverse events. In the event of suspected study drug related toxicity or overdose;

administration of ruxolitinib should be dose reduced or held according to the treating physician's judgement. Ideally an alternative therapy should be sourced rather than reduce the dose of ruxolitinib. For additional information, please refer to the ruxolitinib summary of product characteristics (SmPC).

7.9 PROHIBITED MEDICATION

The following medications are prohibited until treatment discontinuation:

- Concomitant use of another JAK inhibitor

For additional information, please refer to the ruxolitinib SmPC.

7.10 CONCOMITANT MEDICATION

The metabolism of ruxolitinib is affected by CYP3A4 inducers and inhibitors and dual inhibitors of CYP2C9 and CYP3A4 enzymes. Strict attention to this detail is required; please refer to permitted and prohibited concomitant medication as detailed in the SmPC. Patients should not receive any other JAK2 inhibitor whilst on the trial.

All medications, procedures, and significant non-drug therapies (including physical therapy and blood product support) administered after the participant was enrolled into the study must be recorded on the Case Report Forms. Each concomitant drug must be individually assessed against all exclusion criteria/prohibited medication.

7.11 SUPPLY OF STUDY TREATMENT

Ruxolitinib will be provided free of charge by Novartis at the following strengths: 5 mg, 10 mg, and 20 mg.Each tablet contains 5 mg, 10mg, or 20mg ruxolitinib (as phosphate).The tablets should not be stored above 30^0C.PVC/PCTFE/Aluminium blister packs containing 14 or 56 tablets or multipacks containing 168 (3 packs of 56) tablets.

Fostamatinib will be provided free of charge by Rigel at the following strengths: 100 mg and 150 mg. Each film-coated tablet contains either 126.2 mg or 189.3mg of fostamatinib disodium hexahydrate equivalent to 100 mg and 150mg of fostamatinib respectively. This medicinal product does not require any special temperature storage conditions. It should be stored in the original package to protect from moisture and keep the bottle tightly closed.

The local trial pharmacist will be responsible for maintaining and updating the drug accountability log in the MATIS pharmacy file which will be used to monitor compliance. All unfinished bottles will be returned to the trial pharmacist who will count and document any unused medication. All IMPs can then be destroyed in accordance with local pharmacy practice and this will be documented on the drug destruction log in the hospital pharmacy file

7.12 TREATMENT DURATION

The planned duration of treatment is 14 days. Participants may be discontinued from treatment earlier due to unacceptable toxicity or disease progression which will be recorded as a Serious Adverse Event (SAE). If participant is being discharged prior to Day 14 they will go home and continue with the treatment plan.

8 PHARMACOVIGILANCE

8.1 DEFINITIONS

Adverse Event (AE): any untoward medical occurrence in a patient or clinical trial subject administered a medicinal product and which does not necessarily have a causal relationship with this treatment. *An AE can therefore be any unfavourable and unintended sign (including an abnormal laboratory finding), symptom, or disease temporally associated with the use of an investigational medicinal product (IMP), whether or not considered related to the IMP.*

Adverse Reaction (AR): all untoward and unintended responses to an IMP related to any dose administered. *All Aes judged by either the reporting investigator or the sponsor as having reasonable causal relationship to a medicinal product qualify as adverse reactions. The expression reasonable causal relationship means to convey in general that there is evidence or argument to suggest a causal relationship.*

Unexpected Adverse Reaction: an AR, the nature or severity of which is not consistent with the applicable product information (e.g. investigator's brochure for an unapproved investigational product or SmPC for an authorised product*). When the outcome of the adverse reaction is not consistent with the applicable product information this adverse reaction should be considered as unexpected. Side effects documented in the SmPC which occur in a more severe form than anticipated are also considered to be unexpected.*

Serious Adverse Event(SAE) or **Serious Adverse Reaction(SAR):** any untoward medical occurrence or effect that at any dose:

- **Results in death.**

- **Is life-threatening** – *refers to an event in which the subject was at risk of death at the time of the event; it does not refer to an event which hypothetically might have caused death if it were more severe.*
- **Requires hospitalisation, or prolongation of existing inpatients' hospitalisation.**
- **Results in persistent or significant disability or incapacity.**
- **Is a congenital anomaly or birth defect.**

Medical judgement should be exercised in deciding whether an AE/AR is serious in other situations. Important AE/Ars that are not immediately life-threatening or do not result in death or hospitalisation but may jeopardise the subject or may require intervention to prevent one of the other outcomes listed in the definition above, should also be considered serious.

Suspected Unexpected Serious Adverse Reaction (SUSAR): any suspected adverse reaction related to an IMP that is both unexpected and serious.

8.2 CAUSALITY

Most adverse events and adverse drug reactions that occur in this study, whether they are serious or not, will be expected treatment-related toxicities due to the drugs used in this study. The assignment of the causality should be made by the investigator responsible for the care of the participant using the definitions in the table below. If any doubt about the causality exists, the local investigator should inform the study coordination centre who will notify the Chief Investigators. The pharmaceutical companies and/or other clinicians may be asked to advise in some cases. In the case of discrepant views on causality between the investigator and others, all parties will discuss the case. In the event that no agreement is made, the MHRA will be informed of both points of view.

Table 6. Defining causality

Relationship	Description
**Unrelated**	There is no evidence of any causal relationship
**Unlikely**	There is little evidence to suggest there is a causal relationship (e.g. the event did not occur within a reasonable time after administration of the trial medication). There is another reasonable explanation for the event (e.g. the participant's clinical condition, other concomitant treatment).
**Possible**	There is some evidence to suggest a causal relationship (e.g. because the event occurs within a reasonable time after administration of the trial medication). However, the

	influence of other factors may have contributed to the event (e.g. the participant's clinical condition, other concomitant treatments).
**Probable**	There is evidence to suggest a causal relationship and the influence of other factors is unlikely.
**Definitely**	There is clear evidence to suggest a causal relationship and other possible contributing factors can be ruled out.
**Not assessable**	There is insufficient or incomplete evidence to make a clinical judgement of the causal relationship.

8.3 SAE GRADING

Grade 1: Mild; asymptomatic or mild symptoms; clinical or diagnostic observations only; intervention not indicated.

Grade 2: Moderate; Minimal, local, or non-invasive intervention indicated; limiting age appropriate instrumental activities of daily living (ADL).

Grade 3: Severe or medically-significant but not immediately life-threatening; hospitalisation or prolongation of hospitalisation indicated; disabling; limiting self-care ADL.

Grade 4: Life-threatening consequences; urgent intervention indicated.

Grade 5: Death related to AE.

8.4 REPORTING PROCEDURES

All adverse events should be reported. Depending on the nature of the event the reporting procedures below should be followed. Any questions concerning adverse event reporting should be directed to the study coordination centre in the first instance. A flowchart is given below to aid in the reporting procedures.

Non serious AR/Aes

All such toxicities, whether expected or not, should be recorded in the toxicity section of the relevant case report form and sent to the study coordination centre within one month of the form being due.

Serious AR/Aes

Fatal or life-threatening SAEs and SUSARs should be reported on the day that the local site is aware of the event. The SAE form asks for nature of event, date of onset, severity, corrective therapies given, outcome and causality (i.e. unrelated, unlikely, possible, probably, definitely). The responsible investigator should sign the causality of the event. Additional information should be sent within 5 days if the reaction has not resolved at the time of reporting.

SAEs

An SAE form should be completed and faxed to the study coordination centre for all SAEs within 24 hours. However, relapse and death due to <condition>, and hospitalisations for elective treatment of a pre-existing condition do not need reporting as SAEs.

SUSARs

In the case of suspected unexpected serious adverse reactions, the staff at the site should:

Complete the SAE case report form & send it immediately (within 24 hours, preferably by fax), signed and dated to the study coordination centre together with relevant treatment forms and anonymised copies of all relevant investigations.

Or

Contact the study coordination centre by phone and then send the completed SAE form to the study coordination centre within the following 24 hours as above.

The study coordination centre will notify the MHRA, REC and the Sponsor of all SUSARs occurring during the study according to the following timelines; fatal and life-threatening within 7days of notification and non-life threatening within 15 days. All investigators will be informed of all SUSARs occurring throughout the study. Local investigators should report any SUSARs and /or SAEs as required by their Local Research Ethics Committee and/or Research & Development Office.

8.5 COVID-19 CONSIDERATIONS

Recording Suspected Serious Adverse Reactions

The focus is on those events that, based on a single case, are highly likely to be related to the study medication. Examples include anaphylaxis, Stevens Johnson Syndrome, or bone marrow failure, where there is no other plausible explanation. Any SAE that is believed with a reasonable probability to be due to one of the study treatments will be considered a Suspected Serious Adverse Reaction (SSAR). In making this assessment, there should be consideration of the probability of an alternative cause (for example, COVID-19 itself or some other condition preceding randomisation), the timing of the event with respect to study treatment, the response to withdrawal of the study treatment, and (where appropriate) the response to subsequent re-challenge. All SSARs should be reported by telephone to the Central Coordinating Office and recorded on the study IT system immediately.

Central assessment and onward reporting of SUSARs

Clinicians at the Central Coordinating Office are responsible for expedited review of reports of SSARs received. Additional information (including the reason for considering it both serious and related, and relevant medical

and medication history) will be sought. The focus of SUSAR reporting will be on those events that, based on a single case, are highly likely to be related to the study medication. To this end, anticipated events that are either efficacy endpoints, consequences of the underlying disease, or common in the study population will be exempted from expedited reporting. Thus, the following events will be exempted from expedited reporting:

(i) Events which are the consequence of COVID-19; and

(ii) Common events which are the consequence of conditions preceding randomisation.

Any SSARs that are not exempt will be reviewed by a Central Coordinating Office clinician and an assessment made of whether the event is “expected” or not (assessed against the relevant Summary of Product Characteristics or Investigator Brochure). Any SSARs that are not expected would be considered a Suspected Unexpected Serious Adverse Reaction (SUSAR).All confirmed SUSARs will be reported to the Chair of the DMC and to relevant regulatory authorities, ethics committees, and investigators in an expedited manner in accordance with regulatory requirements.

Recording other Adverse Events

In addition to recording Suspected Serious Adverse Reactions, information will be collected on all deaths and efforts will be made to ascertain the underlying cause. Other non-serious adverse events will not be recorded. It is anticipated that for some sub-studies, more detailed information on adverse events (e.g. through linkage to medical databases) or on other effects of the treatment (e.g. laboratory or radiological features) will be recorded and analysed but this is not a requirement of the core protocol.

Figure 3. Safety Reporting Overview

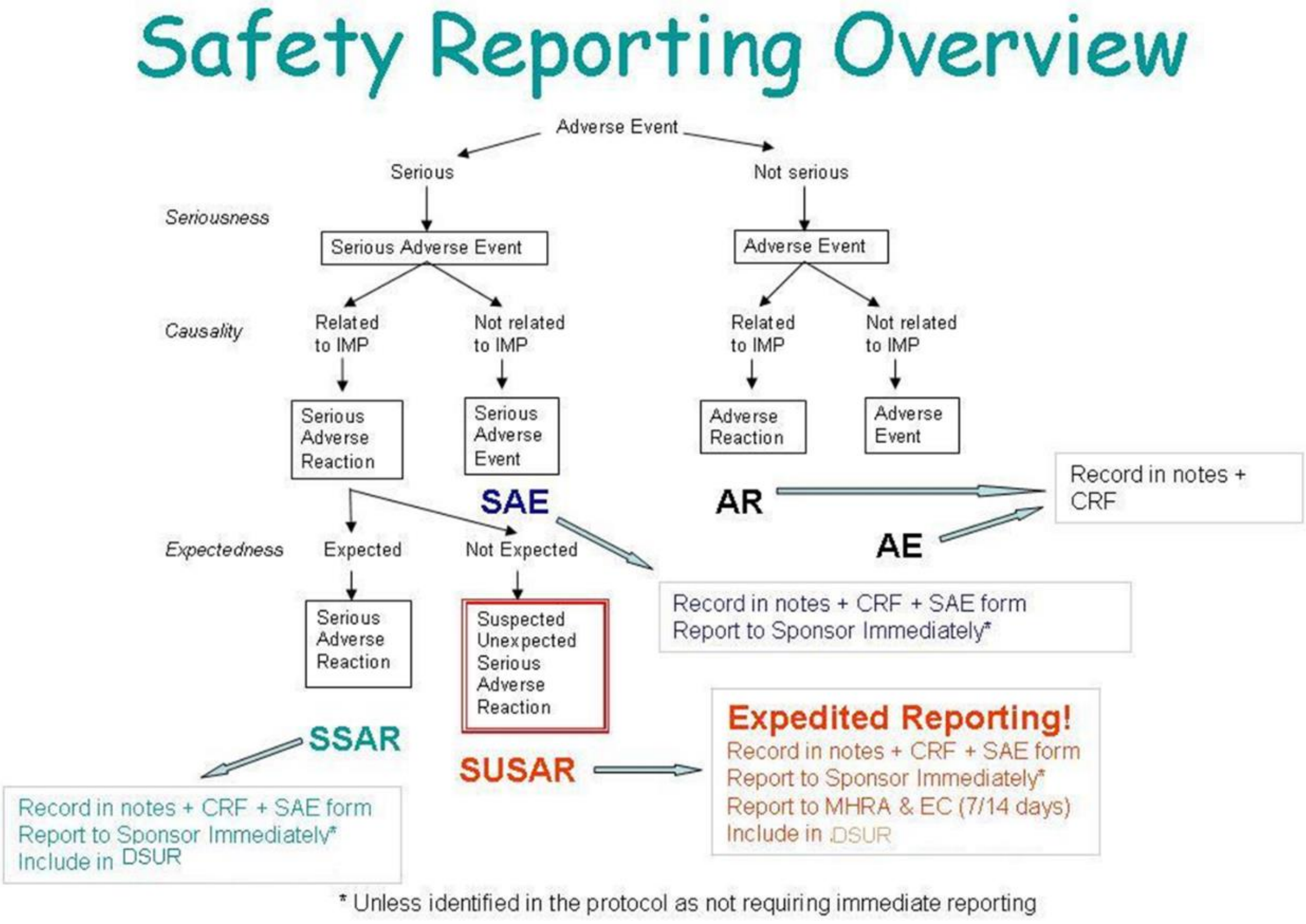

Contact details for reporting SAEs and SUSARs

rgit.ctimp.team@imperial.ac.uk

CI email n.cooper@imperial.ac.uk

Please send SAE forms to: rgit.ctimp.team@imperial.ac.uk and **n.cooper@imperial.ac.uk**
Tel:+ 44 (0)20 7594 9480(Mon to Fri 09.00 – 17.00)

9 ASSESSMENT AND FOLLOW-UP

9.1 COLLECTING FOLLOW-UP INFORMATION

The following information will be ascertained at the time of death or discharge or at 28 days after randomisation (whichever is sooner):

- Vital status (alive / dead, with date and presumed cause of death, if appropriate)
- Hospitalisation status (inpatient / discharged, with date of discharge, if appropriate)
- Use of ventilation (with days of use and type, if appropriate)
- Use of renal dialysis or haemofiltration

This information will be obtained and entered into the web-based IT system by a member of the hospital clinical or research staff. Follow-up information is to be collected on all study participants, irrespective of whether or not they complete the scheduled course of allocated study treatment. Study staff will seek follow-up information through various means including medical staff, reviewing information from medical notes, routine healthcare systems, and registries.

9.2 DURATION AND MODE OF FOLLOW-UP

All randomised participants are to be followed up until death, discharge from hospital or 28 days after randomisation (whichever is sooner). It is recognised that in the setting of this trial, there may be some variability in exactly how many days after randomisation, information on disease status is collected. This is acceptable and will be taken account of in the analyses and interpretation of results, the principle being that some information about post-randomisation disease status is better than none.

Where discharge occurs before 14 days, study medication will be sent home with patient. The study team will send a pre-paid envelope to the patients to return any unused medication and empty bottles if patients are not able to attend Day 14 and Day 28 visits in person.

Where discharge occurs before 28 days, patients will be invited to return weekly for monitoring of blood tests to confirm continued recovery from illness. Where this is not possible, monitoring of clinical status will be achieved by telephone.

Longer term (up to 10 years) follow-up will be sought through linkage to electronic healthcare records and medical databases including those held by NHS Digital, Public Health England and equivalent bodies, and to relevant research databases (e.g. UK Biobank, Genomics England).

Table 7. Schedule of events

	Screening	Baseline				Last visit
Study Day	***-6 to 0***	***0***	***1⁺***	***7#***	***14#***	***28#***
Time window		*-2 days*	*+ 1 day*	*±3 days*	*±3 days*	*±3days*
Informed consent	x					
Inclusion/Exclusion criteria	x	x				
Demographic data	x					
Medical history including major comorbidities	x					
Physical exam	x					
COVID-19 diagnosis, duration and severity	x					
Pregnancy test	x					
Vital signs	x	x	x	x	x	x
NEWS score	x	x	x	x	x	x
Inflammatory markers (CRP, D-dimer, LDH, ferritin, full blood count)		x	x	x	x	x
PK levels*					x	x
Full blood count(FBC)¶		*Daily while on CYP3A4 inhibitor*				
Coagulation, Chemistry, Ferritin, Troponin, Procalcitonin	x	x	x	x	x	x
FiO_2	x	x	x	x	x	x
SpO_2 and/or PaO_2	x	x	x	x	x	x
Randomisation		x				
Weight and Estimated height		x				
Safety monitoring of liver and renal function	x	x	x	x	x	x
Length of stay						
Serious Adverse Events	x	x	x	x	x	x
Concomitant medications	x	x	x	x	x	x
Serum		x		x	x	x
Plasma		x		x	x	x
RNA in PAXgene tube		x		x	x	x
PBMC		x		x	x	x
Nasosorption biomarkers		x		x	x	x

⁺ data that is routinely collected for clinical purposes will be recorded and a visit is not required

in the event that the patient recovers from COVID19 and is discharged, these visits will not be mandated. Instead a visit should occur on the date of discharge. Where possible, patients will be contacted by telephone at day 7, 14 and 28 and invited to attend for blood tests to monitor resolution of illness.

*Ruxolitinib dose for these patients will be assessed after 10 dialysis patients complete Day 14 through pharmacokinetics (PK) levels which will be assessed.

¶For patients randomized to ruxolitinib and prescribed a strong CYP3A4 inhibitor or dual CYP3A4/CYP2C9 inhibitor. Please refer to Section 7.8 for details.

9.1 INCIDENTAL FINDINGS

If there are any incidental findings of any relevance to the participants at any time during their participation in the study, then these will be explained to them. Any incidental findings will be recorded in the clinical notes and will be documented in GP and discharge letters.

9.2 LOST TO FOLLOW-UP

The study team will make every reasonable effort to contact participants who have been discharged while on the study to complete the necessary protocol assessments. If any participants are lost to follow-up then the entire data set will be analysed as per the ITT method.

9.3 END OF TRIAL

The end of the scheduled treatment phase is defined as the date of the last Follow-up visit of the last participant. The end of the study is the date of the final data extraction from NHS Digital (anticipated to be 10 years after the last patient is enrolled).

10 TRIAL MANAGEMENT

The trial will be coordinated by a Central Coordinating Office within the Department of Haematology staffed by members of the Non-malignant Haematology Clinical Trials Unit.

10.1 TRIAL MANAGEMENT GROUP

A Trial Management Group (TMG) will be established, and will include the Chief Investigator Dr Nichola Cooper, the trial statistician and the trial coordinator. Key trial personnel will be invited to join the TMG as appropriate to ensure representation from a range of professional groups. Notwithstanding the legal obligations of the Sponsor and Chief Investigator, the TMG will be responsible for the day-to-day running and management of the trial and will meet by teleconference or in-person as required. Please refer to the TMG Charter for details.

10.2 TRIAL STEERING COMMITTEE (TSC)

The Trial Management Group will also serve as the Trial Steering Committee (TSC) and will provide overall trial supervision and provide advice. The ultimate decision for the continuation of the trial lies with the TSC. The TSC will meet at least once a year or more often if required. Please refer to the TSC Charter for details.

10.3 DATA MONITORING COMMITTEE

During the study, interim analyses of all study data will be supplied in strict confidence to the independent DMC, which will comprise an independent statistician. The DMC will request such analyses at a frequency relevant to the emerging data from this and other studies.
The DMC will independently evaluate these analyses and any other information considered relevant. The DMC will determine if, in their view, the randomised comparisons in the study have provided evidence on mortality that is strong enough (with a range of uncertainty around the results that is narrow enough) to affect national and global treatment strategies. In such a circumstance, the DMC will inform the TSC who will make the results available to the public and amend the trial arms accordingly. Unless this happens, the Steering Committee, Chief Investigator, study staff, investigators, study participants, funders and other partners will remain blind to the interim results until 28 days after the last patient has been randomised for a particular intervention arm (at which point analyses may be conducted comparing that arm with the SOC arm).

The Data Monitoring Committee (DMC), will give advice on whether the accumulated data from the trial, together with the results from other relevant research, justifies the continuing recruitment of further patients. Meetings may be called if recruitment is much faster than anticipated and the DMC may, at their discretion, request to meet more frequently or continue to meet following completion of recruitment. An emergency meeting may also be convened if a safety issue is identified. The findings will be conveyed to the MHRA, funders, and/or sponsors as applicable. The DMC may consider recommending the discontinuation of the trial if the recruitment rate or data quality are unacceptable or if any issues are identified which may compromise patient safety.

Please refer to the DMC Charter for details.

11 MONITORING

11.1 RISKS AND BENEFITS

There is evidence that blockade/inhibition of the JAK/STAT pathway could have a beneficial effect on the CRS and the course of severe respiratory disease/ARDS in patients with COVID-19. However, ruxolitinib has not previously been studied in patients with COVID-19 pneumonia. Therefore, it is unknown as to whether there will be a benefit for patients being treated with ruxolitinib in this disease.

Important identified and potential risks from ruxolitinib clinical development and post authorization experience to date include: infections, tuberculosis, use in patients with hepatic impairment and with moderate or severe renal failure or end stage renal failure, elevated transaminases, bleeding, progressive multifocal leukoencephalopathy, adverse events after discontinuation of ruxolitinib, non-melanoma skin cancer, hepatitis B reactivation, and developmental toxicity.

Potential risks for research participants

No disadvantages are anticipated for research participants emanating from taking part in the study.

Potential benefits for research participants

There is no direct clinical benefit for research participants, but the information generated by this study may benefit future patients.

11.2 MONITORING AT STUDY COORDINATION CENTRE

Staff at the Non-malignant Haematology Clinical Trials Unit will review Case Report Form (CRF) data for errors and missing key data points. The trial database will also be programmed to generate reports on errors and error rates. Essential trial issues, events and outputs, including defined key data points, will be detailed in the MATIS trial Data Management Plan.

11.3 MONITORING AT LOCAL SITES

The frequency, type and intensity of routine and triggered on-site monitoring will be detailed in the MATIS Quality Management and Monitoring Plan (QMMP). The QMMP will also detail the procedures for review and sign-off of monitoring reports. In the event of a request for a trial site inspection by any regulatory authority the Sponsor must be notified as soon as possible.

11.4 DATA AND SAFETY MONITORING

Follow-up safety assessment will occur at day 7, 14 and 28 days. Serious adverse events (SAEs) will be collected and reported in an expedited fashion. The requirements for collection of AEs is as follows:

- All SAEs
- All non-serious AEs
- All reports of IMP exposure during pregnancy
- All reports of misuse and abuse of an IMP, other medication errors and uses outside of what is foreseen in the protocol (irrespective if a clinical event has occurred

12 ETHICAL CONSIDERATIONS AND REGULATORY COMPLIANCE

This study will be carried out in compliance with the protocol and the principles of Good Clinical Practice (ICHGCP) & the Medicines for Human Use (Clinical Trials) Regulations 2004 and Amendment Regulations 2006 and is registered under the General Data Protection Regulation. The Investigator should ensure that all persons assisting with the study are adequately informed about the protocol-related duties and maintain a list of Sub-investigators and other appropriately qualified persons to whom he or she has delegated significant study-related duties. The Investigator is responsible for keeping a record of all subjects who sign an informed consent document and are screened for entry into the study. All versions of trial documents will have the relevant ethical approvals.

12.1 CTA

This study has Clinical Trials Authorisation from the UK Competent Authority; MHRA.
Reference:19174/0421/001-0001

12.2 ETHICS APPROVAL

The Study Coordination Centre has obtained approval from the Surrey Research Ethics Committee (REC) and Health Regulator Authority (HRA). The study must also receive confirmation of capacity and capability from each participating NHS Trust before accepting participants into the study or any research activity is carried out. The study will be conducted in accordance with the recommendations for physicians involved in research on human subjects adopted by the 18th World Medical Assembly, Helsinki 1964 and later revisions.

Protocol Amendments

All protocol amendments will be approved by the sponsor prior to submission to the Ethics Committee and implementation.

12.3 CONSENT

Informed consent should be obtained from each patient before enrolment into the study. However, if the patient lacks capacity to give consent due to the severity of their medical condition (e.g. acute respiratory failure or need for immediate ventilation), then consent may be obtained from a relative acting as the patient’s legally designated personal representative. Further consent will then be sought with the patient if they recover sufficiently.

Due to the poor outcomes in COVID-19 patients who require ventilation (>90% mortality in one cohort5), patients who lack capacity to consent due to their disease, and for whom a relative to act as the legally designated representative is not immediately available, randomisation and consequent treatment will proceed with consent provided by a treating clinician (independent of the clinician seeking to enrol the patient) who will act as the legally designated professional representative. Consent will then be obtained from the patient's personal legally designated representative (or directly from the patient if they recover promptly) at the earliest opportunity.

Informed consent can be taken by any competent health care professional delegated by the chief investigator which can include doctors, nurses and research practitioners. If nurses or research practitioners take consent, the most up to date Legal Representative Consent Form (LCRF) should be used with the patient's treating clinician, independent of the study team, to sign as Legal Representative and the nurse/research practitioner as the Person taking consent.

12.4 WITHDRAWAL OF CONSENT

A decision by a participant that they no longer wish to continue receiving study treatment should **not** be considered to be a withdrawal of consent for follow-up. However, participants are free to withdraw consent for some or all aspects of the study at any time if they wish to do so. In accordance with regulatory guidance, de-identified data that have already been collected and incorporated in the study database will continue to be used (and any identifiable data will be destroyed).

12.5 CONFIDENTIALITY

The research team may exchange emails between themselves about the study and its data analysis. Personal addresses, postcodes, faxes, emails or telephone numbers may be used to post study documents to potential participants. The signed consent forms and completed health questionnaires will be stored safely in the locked clinical trials office, in the Hammersmith Hospital. All electronic files created for the study database will be stored in a single NHS computer, access to which is limited by a password. All electronic files/records created for the study will be password protected. The study team will ensure that the confidentiality of participant data is preserved and will only use NHS email accounts (@nhs.net) to communicate for the study. Participant names will not be disclosed and will not appear on any reports produced. The EU General Data Protection Regulation (GDPR) that came into effect on 25 May 2018 defines expanded rights for study patients. The study team will

inform all participants of these rights. Personal data will be kept in line with Imperial College Healthcare NHS Trust policy for ten years.

Representatives of the sponsor will be granted direct access to original medical records for verification of trial participation and data without violating the confidentiality of these records to the extent permitted by the applicable laws and regulations. Identifiable data will be stored in the patients' medical records and NHS computers. Participants will consent to this access by signing the informed consent form.

12.6 INDEMNITY

Imperial College London holds negligent harm and non-negligent harm insurance policies which apply to this study.

12.7 SPONSOR

Imperial College London will act as the Trial Sponsor.

12.8 FUNDING

This study is supported by the NIHR Imperial Biomedical Research Centre. The data will be collected, analysed and published independently of the source of funding; however study outcome results will be shared with Novartis or Rigel following interim analyses and prior to publication.

12.9 AUDITS AND INSPECTIONS

The study may be subject to inspection and audit by Imperial College Healthcare NHS Trust under their remit as sponsor to ensure adherence to GCP. The Chief Investigator will be responsible for the conduct and progress monitoring of the study.

13 PUBLICATIONS AND REPORTS

The Steering Committee will be responsible for drafting the main reports from the study and for review of any other reports. In general, papers initiated by the Steering Committee (including the primary manuscript) will be written in the name of the MATIS Collaborative Group, with individual investigators named personally at the end of the report (or, to comply with journal requirements, in web-based material posted with the report).

The Steering Committee will also establish a process by which proposals for additional publications (including from independent external researchers) are considered by the Steering Committee. The Steering Committee will facilitate the use of the study data and approval will not be unreasonably withheld. However, the Steering

Committee will need to be satisfied that any proposed publication is of high quality, honours the commitments made to the study participants in the consent documentation and ethical approvals, and is compliant with relevant legal and regulatory requirements (e.g. relating to data protection and privacy). The Steering Committee will have the right to review and comment on any draft manuscripts prior to publication.

14 REFERENCES

Elli, E.M., Baratè, C., Mendicino, F., Palandri, F., et al. (2019) Mechanisms Underlying the Anti-inflammatory and Immunosuppressive Activity of Ruxolitinib. Frontiers in Oncology. [Online] 9, 1186. Available from: doi:10.3389/fonc.2019.01186.

Huang, C., Wang, Y., Li, X., Ren, L., et al. (2020) Clinical features of patients infected with 2019 novel coronavirus in Wuhan, China. Lancet (London, England). [Online] 395 (10223), 497–506. Available from: doi:10.1016/S0140-6736(20)30183-5.

Jagasia, M., Perales, M.-A., Schroeder, M.A., Ali, H., et al. (2020) Ruxolitinib for the treatment of steroid-refractory acute GVHD (REACH1): a multicenter, open-label phase 2 trial. Blood. [Online] 135 (20), 1739–1749. Available from: doi:10.1182/blood.2020004823.

Richardson, P., Griffin, I., Tucker, C., Smith, D., et al. (2020) Baricitinib as potential treatment for 2019-nCoV acute respiratory disease. Lancet (London, England). [Online] 395 (10223), e30–e31. Available from: doi:10.1016/S0140-6736(20)30304-4.

Ruan, Q., Yang, K., Wang, W., Jiang, L., et al. (2020) Clinical predictors of mortality due to COVID-19 based on an analysis of data of 150 patients from Wuhan, China. Intensive Care Medicine. [Online] 46 (5), 846–848. Available from: doi:10.1007/s00134-020-05991-x.

Siddiqi, H.K. & Mehra, M.R. (2020) COVID-19 illness in native and immunosuppressed states: A clinical–therapeutic staging proposal. The Journal of Heart and Lung Transplantation. [Online] 39 (5), 405–407. Available from: doi:10.1016/j.healun.2020.03.012.

Singanayagam, A., Glanville, N., Girkin, J.L., Ching, Y.M., et al. (2018) Corticosteroid suppression of antiviral immunity increases bacterial loads and mucus production in COPD exacerbations. Nature Communications. [Online] 9 (1), 2229. Available from: doi:10.1038/s41467-018-04574-1.

Wu, D. & Yang, X.O. (2020) TH17 responses in cytokine storm of COVID-19: An emerging target of JAK2 inhibitor Fedratinib. Journal of Microbiology, Immunology, and Infection. [Online] 53 (3), 368–370. Available from: doi:10.1016/j.jmii.2020.03.005.

PRINCIPALINVESTIGATOR SIGNATURE PAGE

The signature of the below constitutes agreement of this protocol by the signatory and provides the necessary assurance that this study will be conducted at his/her investigational site according to all stipulations of the protocol including all statements regarding confidentiality.

Study Title: **MATIS: Multi-arm trial of Inflammatory Signal Inhibitors for COVID-19**

Address of Institution: ____

____

____

Print Name and Title: ____

Signed: ____

Date: ______________________
